# The Genetic Variation of *RELN* Expression in Schizophrenia and Bipolar Disorder

**DOI:** 10.1371/journal.pone.0019955

**Published:** 2011-05-16

**Authors:** Galit Ovadia, Sagiv Shifman

**Affiliations:** Department of Genetics, The Institute of Life Sciences, The Hebrew University of Jerusalem, Jerusalem, Israel; University of Queensland, Australia

## Abstract

Reelin plays an important role in the development and function of the brain and has been linked to different neuropsychiatric diseases. To further clarify the connection between reelin and psychiatric disorders, we studied the factors that influence the expression of reelin gene (*RELN*) and its different isoforms. We examined the total expression of *RELN*, allelic expression, and two alternative *RELN* isoforms in postmortem brain samples from patients with schizophrenia and bipolar disorder, as well as unaffected controls. We did not find a significant reduction in the total expression of *RELN* in schizophrenia or bipolar disorder. However, we did find a significant reduction of the proportion of the short *RELN* isoform, missing the C-terminal region in bipolar disorder, and imbalance in the allelic expression of *RELN* in schizophrenia. In addition, we tested the association between variation in *RELN* expression and rs7341475, an intronic SNP that was found to be associated with schizophrenia in women. We did not find an association between rs7341474 and the total expression of *RELN* either in women or in the entire sample. However, we observed a nominally significant effect of genotype-by-sex interaction on the variation in microexon skipping. Women with the risk genotype of rs7341475 (GG) had a higher proportion of microexon skipping, which is the isoform predominant in tissues outside the brain, while men had the opposite trend. Finally, we tested 83 SNPs in the gene region for association with expression variation of *RELN*, but none were significant. Our study further supports the connection between *RELN* dysfunction and psychiatric disorders, and provides a possible functional role for a schizophrenia associated SNP. Nevertheless, the positive associations observed in this study needs further replication as it may have implications for understanding the biological causes of schizophrenia and bipolar disorder.

## Introduction

Reelin, the protein encoded by the *RELN* gene, is an extracellular matrix-associated glycoprotein expressed in the developing brain by Cajal–Retzius cells in the marginal zone of the cortex and hippocampus and by the cerebellar granule cells in the in the external granule cell layer. In the adult brain reelin is most abundant in GABAergic neurons [1], [2]. It is involved in neuronal migration and has been linked to processes of synaptic plasticity, learning and memory [3]. *RELN* was initially identified as the gene mutated in mice with an abnormal reeling gait [4]. Mice lacking the reelin protein have a neuroanatomical defect of an inverted cortex [5]. Mutations in humans are associated with an autosomal recessive form of lissencephaly with cerebellar hypoplasia [6]. Mice heterozygote for the *RELN* mutation show subtle behavioral anomalies compared to wild types including a deficit in pre-pulse inhibition, contextual fear conditioning, social interaction and social recognition and deficits in learning tasks [7].

The *RELN* gene contains at least two alternative isoforms that are conserved across different species suggesting that they are functionally important [8]. One of them is an alternative splicing event, involving a microexon of 6 nucleotides long. The inclusion of the microexon is brain specific. The other isoform is produced by alternative polyadenylation that results in a truncated protein lacking the highly basic terminus of the reelin protein [8]. These two alternative transcripts affect the 3′ end part of the *RELN* gene, and so they are expected to have a regulatory role on the efficient activation of downstream signaling [9], [10].

Studies of postmortem brain tissues from schizophrenia patients showed a clear reduction of *RELN* mRNA and protein levels, of up to 50% [11], [12], [13]. A similar reduction is also seen in post-mortem brains from bipolar patients [14], [15]. This reduction of *RELN* expression in schizophrenia and bipolar post-mortem brain tissues was considered among the most consistent molecular findings in these diseases [16]. However, in different studies the reduction in reelin was observed in different regions of the brain and was measured using diverse techniques. Moreover, some have failed to replicate this reduction [17]. The transcription start site and the first exon of *RELN* are GC rich, forming a vast CpG island. The CpG island offers an optional transcription regulation mechanism of *RELN* through DNA methylation of the promoter [18], [19]. It has been proposed that the reduction in reelin expression observed in psychiatric patients is a consequence of hypermethylation of the CpG island [20], [21], but this observation was not confirmed by other studies [22], [23]. Another potential modifier of *RELN* expression is a polymorphic GGC repeat in the gene 5' untranslated region. This repeat was found to affect *RELN* expression in an *in-vitro* study, using a reporter gene assay [24].

The above observations, together with the functions of reelin in neuronal migration and synaptic plasticity, led to the hypothesis that reduced reelin levels may increase susceptibility to psychiatric disorders, and that reelin is important to the pathogenesis of schizophrenia [25]. Consistent with this hypothesis, we have recently reported a genome-wide association of schizophrenia in an Ashkenazi Jewish (AJ) population that showed a sex-specific association between an intronic single nucleotide polymorphism (SNP) in the *RELN* gene and schizophrenia [26]. We pursued one SNP (rs7341475) in *RELN*, showing women-specific association, because it was approaching genome-wide significance in the first sample that we genotyped. We were able to replicate the results in a combined data from four additional populations with an estimated risk of OR = 1.58 (*P* = 8.8×10^−7^; gene-sex interaction: *P* = 1.6×10^−5^). A follow-up study in another independent sample of schizophrenia cases and controls from the Ashkenazi Jewish population replicated the women-specific association with schizophrenia of rs7341475, although with very modest significance [27]. Furthermore, we used a meta-analysis to test the association of rs7341475 with schizophrenia by adding the genotype results of samples published in additional four GWAS of schizophrenia and excluding the initial discovery sample [28]. The association between rs7341475 and schizophre+nia in women, after excluding the data from AJ, was significant (p = 9.0×10^−3^), with a calculated odds ratio (OR) of 1.11, much smaller than the original result. A recent study that looked at intermediate phenotype measures of brain structure, brain function, and gene expression, did not find a significant association with rs7341475 genotypes [29].

To further clarify the connection between *RELN* and psychiatric disorders such as schizophrenia and bipolar disorder we studied the factors that influence the expression of *RELN* and its different isoforms. To do so, we used DNA and RNA from the Stanley Array Collection that includes samples from three diagnostic groups: schizophrenia, bipolar disorder, and unaffected controls. Our aims were to identify genetic variants that influence gene expression or splicing patterns, to try to identify a functional role for rs7341475, and to test for differences in *RELN* expression between cases and controls.

## Results

### *RELN* expression in postmortem brains of schizophrenia and bipolar disorder

**Figure 1:**
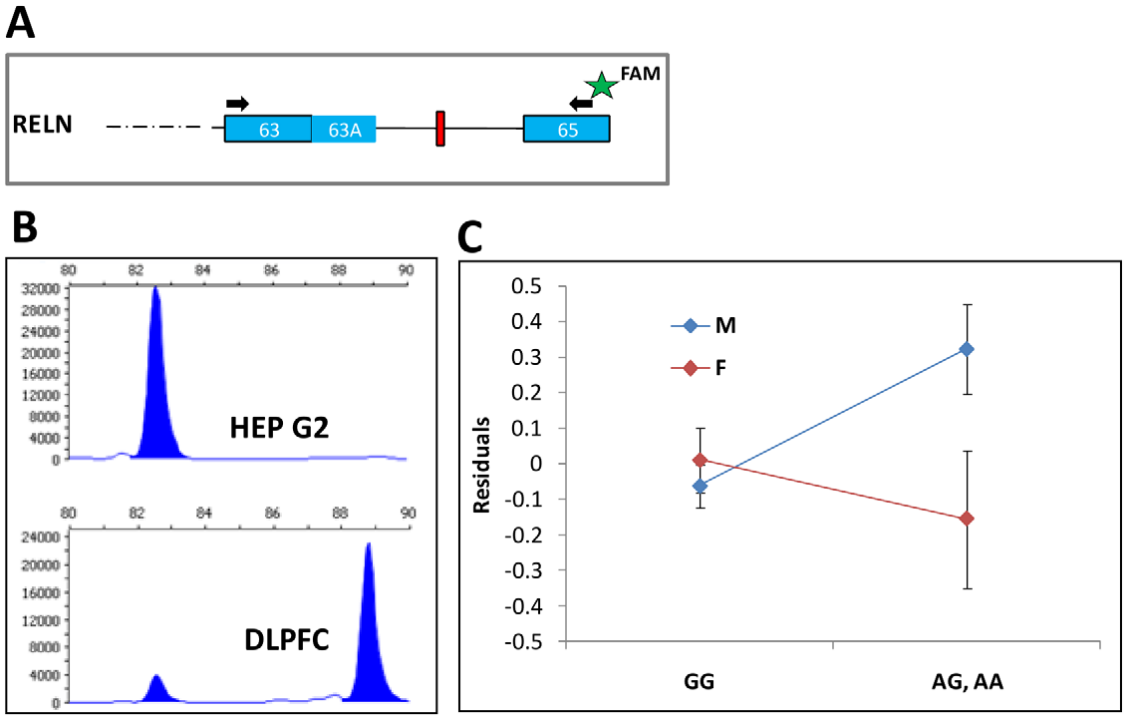
Alternative splicing of microexon (exon #64) in reelin gene. (A) Schematic representation of the last exons of *RELN*. Boxes are for exons and lines are for introns (not to scale). The 6-nucleotides microexon (red box) and the alternative polyadenylation site (63A) are shown. The arrows indicate the positions of PCR primers that were used to measure the alternative splicing of the microexon. The right primer is conjugates to FAM fluorescent dye (represented as a star) to enable detection with a DNA analyzer. (B) Representative results obtained using this assay with RNA from liver cells (HEP G2) and from the dorsolateral prefrontal cortex (DLPFC). In liver cells the microexon is completely skipped, while in brain samples it is mostly retained. (C) The effect of the interaction between rs7341475 genotype and sex on microexon retention. The proportion of microexon retention was first fitted to the level of brain pH. The residuals from the fitted model are plotted on the Y axis and the genotypes of rs7341475 (GG vs AG or AA) on the X axis. An opposite trend is seen in men (n = 61) and women (n = 33): an increase of microexon skipping in women with the GG genotype and in men with the A bearing genotype.

We used DNA and RNA that were extracted from the prefrontal cortex. The samples were from the Stanley Array Collection, which includes postmortem brain tissues from three diagnostic groups: schizophrenia, bipolar disorder, and unaffected controls. We quantified the total and allelic expression of *RELN*, an alternative polyadenylation transcript, and alternative splicing of a microexon. To quantify the total expression of *RELN* and the relative expression of the alternative polyadenylation transcript, we used real-time PCR. On average, 7.8% of transcripts had the alternative polyadenylation site that resulted in a truncated protein. To quantify the proportion of the 6-nucleotide microexon skipping/retention we used PCRs with fluorescent primer and separated the product on a capillary electrophoresis machine (Figure 1A, 1B). Peak heights were used to estimate the proportion of transcripts that retain the microexon. To validate the accuracy and the linearity of this method, we tested a series of dilutions of two synthetic oligos that are similar in sequence and length to the two possible transcripts. A linear relationship (slope = 0.98) with high correlation (R^2^ = 0.99) was observed between the expected values and the measured proportion calculated from peak heights. When used with the brain samples, 83.3% of the full length *RELN* transcripts retained the microexon. In contrast, the microexon was completely absent in hepatocellular carcinoma cell line (HepG2; ATCC HB-8065) that are derived from liver (Figure 1B), consistent with previous findings that this microexon is brain specific [8].

**Figure 2:**
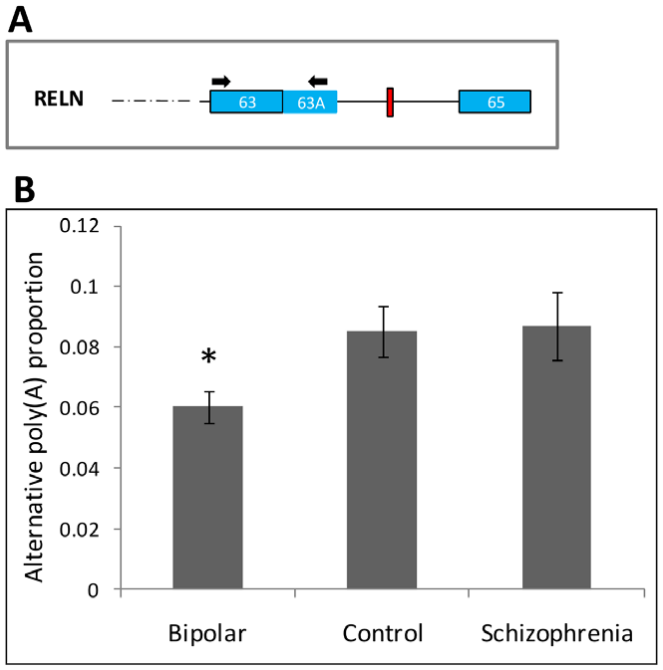
The proportion of transcripts with alternative polyadenylation. (A) Schematic representation of the last exons of *RELN* (as in Figure 1). The alternative polyadenylation site is located in an alternative terminal exon (63A), which could be amplified by PCR with primers as indicated by arrows. (B) The proportion of the short *RELN* isoform, missing the C-terminal region of reelin, in brain samples from bipolar disorder (n = 32), normal control (n = 35) and schizophrenia (n = 35). The bars heights correspond to the mean proportion of transcripts with the alternative polyadenylation event; and error bars are the standard error of the mean. A significant reduction of the proportion of the short *RELN* isoform is observed in bipolar disorder samples (*P* = 0.010).

**Figure 3:**
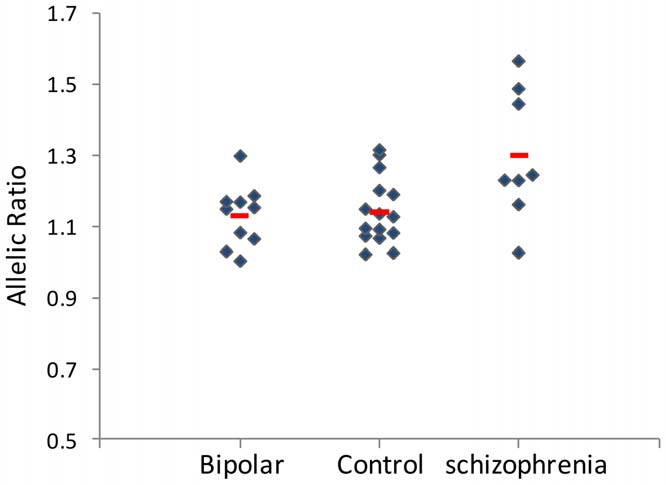
Allelic expression of *RELN* in brain samples from bipolar disorder (n = 10), normal control (n = 15) and schizophrenia (n = 8). Allelic expression was measured six times in each cDNA sample using a coding SNP (rs2229864). The mean allelic ratio (major allele frequency divided by minor frequency) for each informative sample (heterozygote) is presented as a blue diamond. The red horizontal line is the mean for each diagnostic group. Allelic expression imbalance is more pronounced in brain samples from individuals with schizophrenia (*P* = 0.015).

We tested the differences in the expression of *RELN* and its conserved alternative isoforms between the three diagnosis groups. In contrast to previous reports, we did not find a significant reduction in the expression of reelin gene in prefrontal cortex of schizophrenia or bipolar disorder subjects (F = 0.27, df = 2, *P* = 0.76). Correspondingly, non-significant results have been obtained in the analysis of microarray data of the same samples (the Stanley Array Collection), as can be seen at the online genomics database of The Stanley Medical Research Institute (SMRI). Similarly, the proportion of transcripts missing the microexon was not significantly different between cases and controls after controlling for brain PH, which was the most significant confounding factor (F = 12.14, df = 1, *P* = 0.00076). However, the proportion of the alternative polyadenylation transcript (the short isoform) was significantly different between the groups (F = 5.38, df = 2, *P* = 0.0062; Figure 2), being attributed to lower level of the short transcript in the bipolar disorder group (6.0%) relative to control (8.5%, F = 9.0, df = 1, *P* = 0.0040). The difference between schizophrenia (8.7%) and control samples was not significant (F = 0.064, df = 1, *P* = 0.8). The difference between the bipolar disorder samples and normal controls remained significant (F = 6.52, df = 1, *P* = 0.010), after correcting for the most significant confounding factor (post mortem interval, F = 5.12, df = 1, *P* = 0.026). Another expression measure that showed significant differences between the groups was the level of allelic expression imbalance (AEI) (Figure 3). We measured the allelic expression of *RELN* (i.e. the relative expression of the two alleles) using a coding SNP (rs2229864) located in exon 50. Only 33 samples were heterozygotes, hence informative for this assay. The degree of AEI was calculated as the deviation from a balanced expression. The degree of AEI was different among the diagnostic groups (F = 5.12, df = 2, *P* = 0.012), due to a higher level of AEI in schizophrenia samples (F = 7.07, df = 1, *P* = 0.015). Six out of eight informative samples (75%) in the schizophrenia group showed AEI above 1.2, and three of the samples were above 1.4. In contrast, in the control and bipolar samples only four out of 25 (16%) were above 1.2, and none were above 1.4 (Figure 3).

### Correlation between *RELN* expression and genetic variations

Motivated by the evidence that rs7341475 might increase risk for schizophrenia, our first aim was to test for correlation between rs7341475 and *RELN* expression. After controlling for confounding factors, none of the expression measurements were significantly associated with rs7341475 genotypes. However, since the association between rs7341475 and schizophrenia was restricted to women, we tested the effect of genotype by sex interaction on the variation in gene expression. Out of the three expression measurements, there was a nominal significant effect of genotype by sex interaction only with the variation in microexon skipping (F = 5.58, df = 1, *P* = 0.020; Figure 1C), as a result from an opposite trend in men and women. On average, men with the GG genotype showed lower proportion of microexon skipping (15.8%) than men with AA or AG genotypes (22.6%), whereas in women, the GG genotype was associated with a higher proportion of microexon skipping (16.43%) compared to the other genotypes (14.64%).

To screen for other potential *cis*-acting variants, we tested 83 SNPs that were within a 1 Mb window centered at the gene. We tested the association between the genotypes of the 83 SNPs and the variations in total RELN expression, the two alternative isoform and the allelic expression. In addition, we also genotyped the variable GGC repeats in the gene promoter, that was previously shown to have a regulatory effect in a reporter gene expression assay. Similar to a previous report [24], we observed that the majority of alleles (94.8%) are 8 or 10 triplet repeats. We also identified four other long and rare alleles with 12–16 GGC repeats. However, in contrast to the in-vitro study, we did not find an association between *RELN* expression and the GGC repeats. None of the 83 SNPs were significantly associated with variations in *RELN* expression, after correcting for multiple tests.

## Discussion

Reelin plays an important role in the development and function of the brain. As such it was the focus of several studies that examined the variations in the expression of the gene and its possible connection to neuropsychiatric diseases. In this study, we examined four different measures of *RELN* expression: total expression, allelic expression and two alternative *RELN* isoforms. In contrast to some of the previous studies, we did not find a significant reduction in the total expression of *RELN* in postmortem brain samples from patients with schizophrenia or bipolar disorder, even though the sample size was relatively large. It should be noted that previous studies measured Reelin mRNA and protein in several different brain regions and using diverse methods [11], [12], [14], [15], [30]. A previous study that identified a prefrontal cortex reduction in *RELN* expression in patients with schizophrenia and bipolar disorder was performed on a much smaller sample (15 individuals from each group), that were not included in the current study [15]. Thus, the different results may reflect the different methods and samples used in the two studies. Nonetheless, we had two observations that suggest dysfunction of reelin in schizophrenia and bipolar disorder. First, we found a significant reduction of the proportion of the short *RELN* isoform, which is caused by an alternative polyadenylation site, in bipolar disorder samples. Reelin mRNA in the mouse brain consists of 10–25% of this short isoform, missing the C-terminal region (CTR) of reelin [8]. The CTR was found to be required for efficient activation of downstream signaling of reelin [10]. Recently, the short CTR-lacking isoform of Reelin protein was found to be present in the developing brain, and to be secreted from neuronal cells [10]. Although it is clear that the short isoform has a functional role, we do not know what is cause and consequence of the reduction in this isoform in bipolar disorder. The second finding was the imbalance in the allelic expression of *RELN* in postmortem brains of schizophrenic patients, but not in controls or bipolar disorder samples. This finding, although based on a relatively small sample (subset which were informative), suggests that the dysregulation of Reelin identified in schizophrenia is caused by *cis*-acting factors and not by *trans*-acting or external factors that would be expected to influence both alleles equally. The fact that none of the SNPs in the gene region were associated with RELN allelic expression may suggest that the allelic expression imbalance is caused by epigenetic factors. It was recently reported that *RELN* is imprinted in mouse embryonic (E15) brains but not in the adult medial prefrontal cortex. In the embryonic brain, the paternal copy was overexpressed relative to the maternal copy with a ratio of around 1.7 between the paternal and maternal alleles [31]. We do not have the parental genotypes for the samples analyzed in this study; however, one possible explanation for the observed allelic imbalance of *RELN* in schizophrenia might be defects in imprinting erasure in the adult brain. Since the differences in allelic expression between schizophrenia and control subjects are based on a small sample, a further replication study is needed to confirm these results.

Another aim of this study was to test whether rs7341475, an intronic SNP that was found to be associated with schizophrenia in women, has any functional effect on *RELN* expression, and to identify other potential regulatory *cis*-acting variants. To explore the sex-specific genetic effects of rs7341475, we tested the effect of genotype-by-sex (G×S) interaction on the expression of *RELN* and its two conserved isoforms. We found a nominally significant effect of the G×S interaction on the variation in microexon skipping. Consistent with the finding of the genome-wide association study, the risk genotype in women, GG, was associated with higher proportion of microexon skipping, which is the isoform predominant in tissues outside the brain, and with an opposite trend in men. However, if the association of the GG genotype with schizophrenia in women was simply reflected by the higher proportion of microexon skipping in GG carriers we would expect a significant protective effect of this genotype on the risk of schizophrenia in men. Additionally, since we performed several different tests, this result should be interpreted with caution. Finally, we tested 83 SNPs in the gene region for association with expression variation of *RELN*, but none were significant, which may be explained by previous studies suggesting a dominant epigenetic control of RELN expression [18].

In summary, our study further supports the connection between *RELN* dysfunction and psychiatric disorders, and provides a possible functional role for a schizophrenia associated SNP. The allelic expression imbalance in schizophrenia and the lack of association between *RELN* expression and genetic variations, suggests that *cis*-acting factors, such as epigenetic mutations or genetic imprinting defects, are associated with *RELN* dysfunction in schizophrenia and possibly other neuropsychiatric disorders. Additional studies are needed to test the generality of our findings.

## Methods and Materials

### Human postmortem samples

Samples were obtained from postmortem brains that are included in the Stanley Array Collection of the Stanley Medical Research Institute (SMRI). The analyzed sample included 35 individuals with schizophrenia, 32 individuals with bipolar disorder, and 35 unaffected normal controls. For each individual we received DNA and RNA that were extracted from the prefrontal cortex. The experiment was done with coded samples. The diagnostic status, as well as other clinical variables, was provided by SMRI only after we completed the expression assays. The clinical variables included disease status, gender, race, age of onset, postmortem interval (PMI), brain pH, and total brain weight. The demographics details for the Stanley Array samples could be seen at: http://www.stanleyresearch.org/dnn/Portals/0/Stanley/Array%20Collection%20Demographic%20Details%20Chat-Final.pdf. In addition, we obtained genotyping results from Affymetrix SNP 5.0K Array (including 500,000 SNPs) that were previously performed on the same samples [32]. HepG2 human hepatoma cell line (ATCC HB 8065) was from the American Type Culture Collection (Manassas, VA, U.S.A.). The University Committee for the Use of Human Subjects in Research of the Hebrew University of Jerusalem has reviewed and approved the research project and waived the need for consent due to the fact the samples received were collected with consent by the Stanley Medical Research Institute.

### cDNA synthesis

First strand cDNA was generated from around 1 µg of total RNA using random hexamers and SuperScript III First-Strand Synthesis System according to the manufacturer's protocol (Invitrogen). Contamination by genomic DNA (gDNA) was tested using PCR amplification across two exons of the beta-catenin (CTNNB1) gene (primers are listed in Table S1). A larger product was observed, that included the intron, specifically when tested on genomic DNA. PCR reactions were carried out in 10 µl volumes containing 2.5 µl cDNA, 1 µl reaction buffer X10, 1 µl dNTPs (2 mM), 0.04 µl HotStar Taq polymerase (5 unit/µl, Qiagen) and 0.4 µl of each of the sense and antisense primers (10 µM). The reaction included pre-incubation at 95°C for 15 min, followed by 40 cycles of 45 sec at 94°C, 60 sec at 55°C, 30 sec at 72°C and finally, one cycle of 72°C for 5 min. PCR products were separated on 2% agarose gel.

### Real time PCR

Real time PCR in 96-well optical plates (Applied Biosystems) was used to measure the total expression of reelin, as well as the proportion of the alternatively polyadenylated transcript (polyA) (primers are listed in Table S1). Each sample was measured in triplicates. We used ABI PRISM 7900HT Real-Time PCR System with the default thermocycler program for all assays: 10 min of pre-incubation at 95°C followed by 40 cycles of 15 sec at 95°C and one minute at 60°C. Each individual real-time PCR reaction was in 10 µl volumes containing 2.5 µl cDNA, 5 µl ABI - Power Cyber Green Mix and 0.4 µl of the sense and antisense primers (10 µM). The melting curves for each amplicon were inspected to ensure specific single amplification of the PCR product. The Cycle threshold (Ct) was setup manually at the level that reflected the best kinetic PCR parameters. Delta delta Ct method was used in order to estimate the expression of each gene: we subtracted the Ct values of the reelin expression from the Ct values of a reference gene CHL1. CHL1 was selected as a reference gene as it was showing a similar expression in cases and controls in previous studies of the same samples using expression arrays [33]. The delta-Ct value for each sample (based on triplicates average) represented the relative expression of reelin gene. Similarly, we calculated the delta-Ct values for the short alternative polyA transcript by subtracting its Ct values from the Ct value obtained for the total *RELN* expression. In order to determine the efficiency of the PCR amplification, efficiency curves were generated with a series of six dilutions for each reaction (*RELN* efficiency  = 0.99, CHL1 efficiency  = 0.95, PolyA efficiency = 0.95).

### Quantification of the alternatively spliced microexon

In order to quantify the alternatively spliced microexon, which is six nucleotides long, we performed a PCR using two primers flanking the microexon; the sense primer was conjugated with a fluorescence dye (FAM) (primers are listed in Table S1). PCR amplification was performed in a 10 µl volume with 2.5 µl cDNA, 2 µl ReadyMix X5 (LAROVA) and 0.4 µl of the sense and antisense primers. PCR included pre-incubation at 95°C for 2 min, followed by 40 cycles of 15 sec at 94°C, 15 sec at 55°C, 30 sec at 72°C and finally, one cycle of 72°C for 5 min. The PCR products were separated and quantified on ABI PRISM 3730xl DNA Analyzer. The proportion of the shorter transcript (lacking the microexon) was estimated for each sample by relative peak heights that were obtained from the GeneScan software (Applied Biosystems). The proportion values were normalized using the log (base 10) transformation. Each sample was measured in triplicates. In order to validate the accuracy of our method we tested it with known ratios of the two possible transcripts. Synthetic oligos identical by sequence and length to the two amplicons were used to create different ratios. The two oligos were diluted to concentration similar to the one in the cDNA and were mixed in known proportions. The different mixes were amplified by PCR and read in the same manner as described above for cDNA samples (measurements were done in triplicates). The proportion of the shorter oligo was estimated based on the proportion of the peak heights and compared to the expected proportion.

### Genotyping

SNP rs7341475 – We amplified the region containing the SNP rs7341475 [A/G] by PCR. PCR reactions (10 µl) contained 1 µl genomic DNA (4 ng/µl), 1 µl reaction buffer X10, 1 µl dNTPs (2 mM), 0.2 MgCl2 (25 mM), 0.04 µl HotStar Taq polymerase (5 unit/µl, Qiagen) and 0.625 µl of the sense and antisense primers mix (8 µM). The reaction included pre-incubation at 95°C for 15 min, followed by 40 cycles of 60 sec at 94°C, 60 sec at 55°C, 30 sec at 72°C and finally, one cycle of 72°C for 5 min. PCR products were digested with 1 unit of ApoI (New England BioLabs) and run in 3% agarose gel resulting in either three fragments (233+158+30 bp) for the allele A or two fragments (233+188 bp) for the allele G. SNP rs2229864 was genotyped using an Allele Specific Primer Extension (ASPE) assay, and scanned on a BeadXpress reader (Illumina). GGC repeats – we amplified the region containing the triplet repeat in the reelin gene by PCR. The sense primer was conjugated with a fluorescence dye (FAM). PCR amplification was performed in a 10 µl volume with 1 µl genomic DNA (4 ng/µl), 1 µl reaction buffer X10, 1 µl dNTPs (2 mM), 1 µl DMSO 10%, 0.09 µl HotStar Taq polymerase (5 unit/µl, Qiagen) and 0.625 µl of the sense and antisense primers mix (8 µM). We ran the PCR products on ABI PRISM 3730xl DNA Analyzer. We Used the GeneScan software (Applied Biosystems) and determined the number of repeats of alleles by the different fragments sizes.

### Allele Specific Expression

We chose the most common coding SNP in *RELN*. Based on HapMap (CEU), rs2229864 [C/T] has heterozygosity of 44.4%. The SNP is located in the fiftieth exon of the gene. We used an ASPE assay for SNP genotyping using gDNA, as well as for measuring allelic expression in cDNA. Each cDNA sample was measured six times. The assay was comprised of 5 main steps: (1) Amplification of the region containing the measured SNP and products purification (2) Allele specific primer extension for the two alleles (3) Hybridization to holographic beads that are unique for each allele (4) Labeling of the extension sequence (5) Scanning with a BeadXpress reader. Primers were designed for the ASPE assay using the online VeraCode assay designer software: two PCR primers and two ASPE primers. The PCR primers were designed within exon 50 of the reelin gene flanking the SNP region in order to enable amplification of both gDNA and cDNA. The ASPE primers contained one of the alleles [C/T] on the 3' end of the sequence. In addition, each ASPE primer had a different capture sequence of 22 nucleotides that is complementary to the oligos on a selected VeraCode beads (Illumina). The region containing the SNP was amplified by PCR with the following conditions: PCR reactions (10 µl) contained 3 µl gDNA or cDNA (for genotyping or allelotyping respectively), 1 µl reaction buffer X10, 1 µl dNTPs (2 mM), 0.04 µl HotStar Taq polymerase (5 unit/µl, Qiagen) and 0.4 µl of each of the sense and antisense primers (10 µM). The reaction included pre-incubation at 95°C for 15 min, followed by 40 cycles of 45 sec at 94°C, 60 sec at 55°C, 30 sec at 72°C and finally, one cycle of 72°C for 5 min. PCR products were then purified with 1.34 units of Shrimp Alkaline Phosphatase (SAP) and 3.4 units of Exonuclease I (Exo I) and incubated at 37°C for 45 min followed by 15 min at 99°C. During the ASPE reaction, multiple rounds of primer extension were performed, with biotinylated dCTP incorporated into the extension products. Individual ASPE reactions were carried out in 10 µl volume. Each reaction contained 1 µl reaction buffer (X10), 0.5 µl 3 nucleotides mix (dATP,dTTP and dGTP, 100 µM), 0.125 µl biotin 14-dCTP (400 µM), 0.5 µl ASPE primer mix (5 µM, see sequence bellow), 0.1 µl HotStar Taq polymerase (5 Units /µl, Qiagen) and 1.5 µl purified PCR products from the previous step. ASPE reaction included pre-incubation at 95°C for 15 min, followed by 30 cycles of 45 sec at 95°C, 60 sec at 55°C, 30 sec at 72°C. VeraCode beads were kitted into 96-well polypropylene plates (Corning). 8 µl of the ASPE products were transferred into the bead-kitted plate and hybridized with the matching beads. The biotinylated extension products were labeled with streptavidin-Alexa Fluor 647 (Invitrogen). The labeled beads were scanned using a BeadXpress reader.

### Data analysis

Data analysis was performed using the R language and environment for statistical computing (http://www.r-project.org/). Expression data was tested for normal distribution using Shapiro-Wilk normality test. The proportion of microexon skipping and the delta Ct values were normalized using a log transformation. Analysis of Variance (ANOVA) was used to test for differences between the three diagnostic groups, after controlling for significant confounding variables. For analyzing the allelic expression imbalance, we calculated the absolute difference of the proportion of T allele in cDNA from the expected balanced proportion in gDNA. To account for systematic shift in the proportion of the T allele with increase in intensity, we fitted a linear regression model and estimated the slope and intercept for the proportions in gDNA. The expected proportion value for the cDNA was calculated based on this linear model. To test the association between SNPs and expression measurements, we selected SNPs that were in Hardy-Weinberg equilibrium (*P*<0.05/number of SNPs), and with a minor allele frequency above 5%. Homozygote calls were combined with heterozygotes, if the number of homozygotes was equal or lower than five. To test for association between SNPs and allelic expression, we combined the two homozygotes groups together and tested the differences in the allelic expression imbalance for each SNP between heterozygotes and homozygotes. As we tested 83 different SNPs, we corrected for multiple testing by a permutation test. In the permutation test the expression values were randomly distributed among the subjects, whereas the SNPs distribution was unchanged. We ran the permutations until we observed more than 20 instances with a *P*-value equal or lower than the minimum observed *P*-value. The corrected *P*-value was calculated as 20 divided by the number of permutations.

## Supporting Information

Table S1 The sequences of primers used in this study.
(DOC)
