# Supplementary material for: The Genetic Variation of RELN Expression in Schizophrenia and Bipolar Disorder
Source: PLoS One. 2011 May 16;6(5):e19955. doi: 10.1371/journal.pone.0019955 (PMC3095646; doi:10.1371/journal.pone.0019955)
Supplement: Table S1 — The sequences of primers used in this study. (DOC) [file pone.0019955.s001.doc]

|  | Primer | Sequence | Product length |
| --- | --- | --- | --- |
| Reverse transcription | CTNNB1-F | AGGGCTTACTGGCCATCTTT | 238 bp (gDNA)  151 bp (cDNA) |
|  | CTNNB1-R | GCTAAACGCACTGCCATTTT |  |
| Total RELN expression | RelnExp57-58F | TGAGTTCTCGGGAGGAGAGA | 101 bp |
|  | RelnEXP57-58R | GGCACTTCCCATGAAGAAAA |  |
| Alternative PolyA | Reln63A-F | CCTTCCTGAAATGGACATGG | 184 bp |
|  | Reln63A-R | GGACTTCACACAAGCTCAGAGA |  |
| CHL1 expression | RT-chl1ex2.3-F | GTCAAAAGTCCAAGTTGCCTTTC | 102 bp |
|  | RT-chl1ex2.3-R | TTGCCATCCTTAGTCCACGAA |  |
| Microexon splicing | Microexon64-F | AGCCCATGTTGTCGTGAAA | 91 or 85 bp |
|  | Microexon64-R | ATGATCAATGGGCTTTGGAC |  |
| Rs7341475 genotyping | Rs7341475-F | TGCTATGCACTTCATCACGA | 421 bp |
|  | Rs7341475-R | CATGATCCACTCGCCTCA |  |
| GGC allelotyping | GGC-F | CGCCTTCTTCTCGCCTTCTC |  |
|  | GGC-R | CGAAAAGCGGGGGTAATAGC |  |
| ASPE – flanking SNP | Rs2229864-F | GGCTCTGCTGACCAAAG |  |
|  | Rs2229864-R | CATGTCAAAGGCGATCCTC |  |
| ASPE | *ASPE_2229864C_208 | AGTGCCGGTATGATCGCTAACCCAGTACCCCAGCAC |  |
|  | *ASPE_2229864T_42 | GGCACTTCGCACGGGTTACGAACAGTACCCCAGCAT |  |

*ASPE primers include capture sequences to illumine beads VC-301-5634 (Illumi code: 208) and VC-301-5440 (Illumi code: 42).
